# Perceived stress and risky driving among emergency medical services personnel: parallel mediation effects of cognitive failures and emotional exhaustion

**DOI:** 10.3389/fpubh.2026.1827749

**Published:** 2026-04-14

**Authors:** Wejdan Alasqah, Amr Noureldin

**Affiliations:** 1 Department of Business Administration, College of Business and Economics, Qassim University, Buraydah, Saudi Arabia; 2 Department of Business Administration, Faculty of Administrative and Human Sciences, Buraydah Colleges, Buraydah, Saudi Arabia; 3 Department of Business Administration, Faculty of Graduate Studies, Sinai University, Arish, Egypt

**Keywords:** cognitive failures, emergency medical services, emotional exhaustion, perceived stress, risky driving

## Abstract

**Background:**

Risky driving behavior (RDB) while ambulances are engaged in medical emergency services comprises a critical occupational risk as well as a public-safety threat, but the mechanisms underlying such behaviors influenced by perceived stress among emergency medical services (EMS) personnel remain poorly articulated. Based on transactional stress appraisal theory and conservation of resources theory, this study investigated the mediating role of cognitive failures and emotional exhaustion between perceived stress and risky driving.

**Methods:**

A cross-sectional survey was carried out among 400 frontline emergency medical services personnel, including ambulance drivers, emergency medical technicians, and paramedics with recent experience in driving ambulances on duty in Saudi Arabia. Partial least squares structural equation modeling was used to analyze data.

**Results:**

Perceived stress directly did not significantly affect risky driving. But perceived stress positively affected cognitive failures and emotional exhaustion. Cognitive failures and emotional exhaustion, in consequence, had strong positive effects on risky driving. Mediation analysis also indicated specific indirect effects of perceived stress on risky driving through cognitive failures and emotional exhaustion, with both counting as full mediations. The control variables listed were not significant.

**Conclusion:**

Perceived stress affects risky driving behavior among emergency medical services personnel not directly but through both cognitive and affective pathways that are parallel to each other. Stress may also promote risky driving in particular through attention lapses, action and memory failures, stressful task conditions leading to emotional depletion, etc. These findings are an expansion of the understanding of driving safety in emergency medical services and emphasize that to enhance safe driving during ambulance operations, it is vital to address not just stress itself but also the cognitive and emotional fallout from stress.

## 1 Introduction

Emergency medical services (EMS) are a type of safety-critical system that operates in an environment characterized by urgency, unpredictability, high workload, and emotional demand. In this frame, ambulance driving is not just an operational activity for routine transport; rather, it is a complex operational task that needs continuous attention along with expedient judgment and safe behavioral control under pressure (1). Recent evidence indicates that burnout, stress, fatigue, poor quality of sleep, and other issues affect the psychological health of emergency services staff providing prehospital care and increase occupational and patient-safety hazards (2); however, ambulance personnel—who are central to prehospital care in times of emergencies—have been under-researched compared to other professionals working earlier/higher in care (3).

Risky driving behavior during ambulance duty may be an upstream result of perceived stress. Research in professional driving suggests that stressful working conditions may affect safe driving; e.g., Amoadu et al. identified similar effects among bus drivers in Ghana, showing that increasing job demands and job insecurity indirectly increased safety incidents through fatigue driving (4). In emergency medical service settings, clinicians’ wellbeing has important implications for occupational and patient safety, as shown by Tikkanen et al. in their scoping review of prehospital emergency medical services (2). Consistently, Caponnetto et al. reported that perceived stress was a prominent clinical psychological factor in emergency driving settings in a cross-sectional study of ambulance drivers (3), and Zhang et al. reported higher burnout and poorer sleep quality among ambulance drivers with occupational stress (5). Collectively, these findings indicate that perceived stress may increase the likelihood of high-risk driving behavior during ambulance duty.

However, the relationship between perceived stress and unsafe driving is probably not direct. Transactional stress appraisal theory states that stress is also generated when individuals perceive demands as exceeding available coping resources; while conservation of resources theory argues that sustained demands reduce valued cognitive and emotional resources (6, 7). In safety-critical work, such depletion is likely to undermine sustained attention, executive control, and self-regulation. In the EMS setting, this means perceived stress may translate into unsafe driving via workplace cognitive failures and emotional fatigue rather than a direct relationship between stress and behavior (8, 9).

Both mechanisms are relevant for ambulance operations. Specifically, lapses in attention regulation and concentration have also been strongly associated with automated responses such as distracted driving, aggressive driving, driving incidents, and driving errors (10–12). Emotional exhaustion has also been linked with driving anger, fatigue-related driving, driving errors, and a wider set of unsafe occupational driving behavior (13, 14). Due to the time pressure, multitasking needs, traumatic exposure and prolonged operational strain that EMS personnel work under, both cognitive lapses and emotional drainage are likely salient proximal causes of risky driving behavior while on duty (15–18).

Despite these hints, not many studies have looked at a detailed model of risky driving behavior in EMS workers that includes both thinking and feeling aspects in one framework, particularly in Arab and Gulf regions. To fill this gap, the current study investigates whether perceived stress is directly and indirectly influencing risky driving through two parallel mediators among frontline EMS staff in Saudi Arabia: cognitive failures and emotional exhaustion. This study aims to be more definitive than the individual pathways by integrating them through a combined empirical approach that includes stress and behavioral outcomes.

## 2 Literature review and hypothesis

This section presents and details the main theories used to assess and structure various interrelationships between variables, including mediating effects. The literature review is then discussed to establish the hypothesis and its theoretical references.

### 2.1 Theoretical foundations

The proposed model is based on two allied viewpoints. First, transactional stress appraisal theory conceptualizes stress as a result of an individual’s cognitive evaluation of situational demands relative to available coping resources; when demand is assessed to exceed one’s ability to cope, perceived stress emerges and becomes an upstream driver of subsequent cognitive and emotional responses (6, 19). Second, conservation of resources (COR) theory posits that people seek to acquire, build up, and protect valued resources, whereas stress occurs when these resources are threatened, lost, or insufficient to meet ongoing demands; thus, ongoing demands can deplete cognitive and affective resources in a way that increases susceptibility to performance breakdowns and unsafe behavior (7, 20). In safety-critical performance environments, resource depletion is assumed to negatively affect sustained attention and executive function under conditions of high workload (21–23). Building on this reasoning, stress-related depletion may also manifest as workplace cognitive failures—common lapses in attention, memory, and action that are notably costly for work safety (8, 24)—and as emotional exhaustion, a core component of burnout reflecting the depletion of affective energy under persistent demands (9, 25, 26). As a safety-critical task requiring sustained attention, rapid decision-making, and self-regulation in the EMS driving context, duty-related driving is likely to be particularly susceptible to stress-induced cognitive and emotional depletion, such that elevated perceived stress may translate into cognitive failures and emotional exhaustion, thereby increasing the likelihood of behaviors that compromise safe driving (27, 28).

### 2.2 Hypothesis developments

#### 2.2.1 Perceived stress and risky driving

Perceived stress is an important predictor of risky driving, especially in high-demand occupational settings. Evidence from professional driving research suggests that stressful work conditions can undermine safe driving behavior; for example, Amoadu et al. found among bus drivers in Ghana that job demand and job insecurity increased safety incidents indirectly through fatigue driving (4). In emergency medical service settings, Tikkanen et al., in a scoping review of prehospital emergency medical services, concluded that ambulance clinicians’ wellbeing has important implications for occupational and patient safety (2), while Brewster et al. highlighted safety risks faced by paramedics during response and transportation (29). Similarly, Caponnetto et al. reported in a cross-sectional study of ambulance drivers that perceived stress was a salient clinical psychological factor in emergency driving settings (3). Related evidence further shows that occupational stress among ambulance drivers is associated with greater burnout and poorer sleep quality, both of which may impair safe duty performance (5). In addition, higher perceived stress has been linked to more driving errors and violations, as well as higher speeds and fewer positive driving behaviors (30, 31). Based on transactional stress appraisal theory and conservation of resources theory, EMS personnel whose duty demands exceed their available coping resources are more likely to experience reduced attentional control and weakened self-regulation that may together increase engagement in risky driving behavior while on ambulance duty:

*H1:* Perceived stress has a positive association with risky driving.

#### 2.2.2 Perceived stress and cognitive failures

Perceived stress can adversely affect cognitive functioning through the impairment of executive control and reductions of attentional stability and working memory efficacy. El-Sayed et al. reported among postgraduate nursing candidates that higher perceived stress positively predicted cognitive failure and was an important antecedent of forgetfulness and attentional slips (32). Using a large community-based sample of middle-aged and older adults, Campbell et al. demonstrated that subjective cognitive concerns were temporally associated with emotional functioning (33). In the first of these studies that took place in healthcare settings, Parizad et al. reported among emergency department nurses that more frequent cognitive failures were associated with stressful job content, subjective workload and job burnout (34). Mechanistically Carvalho et al. reported poorer working memory performance among stressed adults (35), Salahuddin et al. demonstrated among shift-working healthcare professionals that stress was detrimental to self-discipline over time, indicating exhaustion of the cognitive resources necessary for appropriate monitoring and action control (36). These processes seem particularly relevant in the context of EMS work, where emergency situations are marked by pressure, multitasking and cognitive overload (37). Based on the transactional stress appraisal theory and conservation of resources theory, EMS personnel experience workplace cognitive failures when their duty demands exceed available coping resources due to stress-related depletion in attentional control and self-regulatory capacity. We thus hypothesize that:

*H2:* Perceived stress has a positive association with cognitive failures

#### 2.2.3 Perceived stress and emotional exhaustion

Under prolonged work demands that exceed one’s coping resources, perceived stress is most likely to build as emotional exhaustion. Tatala et al. reported that higher perceived stress was associated with burnout, especially emotional exhaustion, among nurses (38), while Lee et al. demonstrated in acute and critical care settings that burnout was significantly and positively associated with perceived stress (39, 40, 54). This relationship is also relevant to EMS settings, where ambulance personnel are repeatedly exposed to emergencies, shift work, and traumatic events. In this sense, Caponnetto et al. perceived stress as one of the most salient clinical psychological factors among ambulance drivers (3) and Zhang et al. reported a link between occupational stress and worse quality of sleep and burnout in ambulance drivers (5). Altogether, these findings suggest that increased perceived stress could lead to higher emotional exhaustion in EMS personnel. We thus hypothesize that:

*H3:* Perceived stress has a positive association with emotional exhaustion

#### 2.2.4 Cognitive failures and risky driving

Because safe driving relies on sustained attention, working memory, inhibitory control, and adjusting to the ever-changing traffic situation, cognitive failures are likely associated with increased risk during driving. Pergantis et al. demonstrated in a recent overview that executive functions, especially working memory and inhibitory control, are highly interconnected with driving performance as well as risky behaviors; thus, indicating that impairments of these functions may decrease safety while driving under demanding conditions (41). There is also direct empirical evidence for this relationship. Niranjan et al. showed in two separate studies on young adult drivers that cognitive failures predicted a propensity toward distracted driving (12), and Ok et al. reported among Korean taxi drivers that cognitive failure may lead to aggressive driving behavior (13). Similarly, Allahyari et al. found that increased cognitive failure was associated with more driving errors and crashes (10), and Wickens et al. found cognitive failures to predict errors in driving, lapses and violations (11). In the context of occupational driving, cognitive failure has been associated with deliberate violations, unintended violations, slips and mistakes although associations have seemed weaker when considering accidents (42). These mechanisms appear particularly applicable to the work of EMS, who must drive an ambulance with considerable time pressure and perform parallel tasks, experiencing significant cognitive load during emergency response operations and treatment (3, 18, 55, 56). Accordingly, EMS personnel exhibiting high cognitive may be more prone to attentional slips/errors/violations that might increase risky driving behavior during ambulance duty.

*H4:* Cognitive failures have a positive association with risky driving.

#### 2.2.5 Emotional exhaustion and risky driving

Emotional exhaustion is expected to increase risky driving behavior, as the capabilities that underpin sustained attention and restrained driving (i.e., vigilance, accurate judgment of risk/benefit ratios and safe behavioral control) are based on emotional and self-regulatory resources. Li et al. indicated that driving anger, a more proximal construct of risky driving involving violations, aggression, and near-misses (14), was positively associated with emotional exhaustion. In a truck-driver study, Sârbescu et al. reported that driving errors could be predicted by burnout (15), while Amoadu et al. indicated among drivers of heavy goods vehicles in Ghana that burnout was a significant predictor of fatigue driving (16). Likewise, Nguyen et al. reported in a study among bus drivers in Hanoi reported that job burnout was associated with unsafe driving behaviors (17). Although Alonso et al. did not find a significant direct correlation between emotional exhaustion and crash counts among Spanish workers but concluded that emotional exhaustion can negatively affect driving performance and was associated with unfitness to drive (43). These observations are particularly salient across the spectrum of EMS work, where urgency, psychological load, fatigue, and extended operational tempos can drain vigilance and safe behavioral self-governance (2, 3). Consequently, EMS personnel with higher emotional exhaustion are more likely to report risky driving during ambulance duty.

*H5:* Emotional exhaustion has a positive association with risky driving.

#### 2.2.6 Parallel mediation mechanisms

A key idea in transactional stress appraisal theory and conservation of resources theory is that stress is unlikely to translate into risky driving directly; rather, it is expected to operate indirectly through cognitive and affective depletion mechanisms. When EMS workers perceive that job demands exceed their coping capacity, stress may impair concentration and cognitive control, increase the likelihood of cognitive lapses, and gradually deplete emotional resources, resulting in emotional exhaustion. These parallel mechanisms provide a theoretically coherent explanation of how perceived stress may be transformed into hazardous duty-driving behavior. Prior evidence supports this expectation. El-Sayed et al. found that greater stress was associated with higher cognitive failure in a mediational study of postgraduate nursing candidates (32). Likewise, Tatala et al. and Schumann et al. reported positive associations between perceived stress and burnout-related outcomes, including emotional exhaustion, among nurses and undergraduate medical students, respectively (38, 44). Related transport research further suggests that both cognitive failures and emotional exhaustion are associated with unsafe driving outcomes. Niranjan et al. found that increased cognitive failures were associated with heightened distracted driving behaviors (12), while Li et al. and Sârbescu et al. linked emotional exhaustion to driving anger and driving errors, respectively (14, 15). Additional evidence indicates that job stressors may influence safety outcomes indirectly through intermediate depletion processes rather than through direct effects alone. For instance, Amoadu et al. found that among bus drivers high job demand and low job security increased safety incidents indirectly through fatigue driving (4). These concurrent cognitive and affective trajectories are likely to be particularly prominent in an emergency medical services (EMS) context, where urgency, multitasking, emotional burden, and sustained operational demands prevail. Thus, cognitive failures and emotional exhaustion are hypothesized to serve as alternative mediators accounting for the extent to which perceived stress is associated with risky driving behaviors in EMS workers.

*H6a:* Cognitive failures mediate the relationship between perceived stress and risky driving.

*H6b:* Emotional exhaustion mediates the relationship between perceived stress and risky driving.

#### 2.2.7 Control variables

To minimize alternative explanations, the model incorporates specific demographic and occupational characteristics as control variables. Age and experience driving an ambulance may be linked to driving skill, knowledge of how to respond to emergencies, how risky driving is, and how careful drivers are. Weekly working hours and monthly night shifts may indicate workload, fatigue, circadian disruption, and recovery limitations that can affect unsafe driving behaviors while on duty. Also, working night shifts often can make people feel more stressed when they have to work long hours. These variables were incorporated as controls to delineate the impacts of perceived stress, cognitive failures, and emotional exhaustion on risky driving behavior.

## 3 Methods and materials

### 3.1 Measures

The scale (PSS-10) (45) was used to measure perceived stress. The revised 15-item WCFS (8) was used to measure cognitive failures at work. The CBI work-related burnout items (9) were used to measure emotional exhaustion. The leisure-energy item was coded backwards. The occupational driving behavior items (46) were used to measure risky driving (ambulance duty driving). The wording was changed only slightly to fit the EMS context, and the seatbelt item was reverse-coded if you got a “riskier driving” score.

### 3.2 Study designs

This study used a cross-sectional survey and partial least square structural equation modeling (PLS-SEM) via Smart-PLS to investigate the mediating roles of cognitive failures and emotional exhaustion in how perceived stress predicts risky driving. Data were collected from frontline EMS personnel (ambulance drivers, EMTs, and paramedics) working in Saudi Arabia from 12 EMS stations/centers from January to March 2026. Eligibility criteria included having driven an ambulance while on duty in the past 4 weeks. Recruitment used a multi-site strategy, stratified by region and urban–rural setting with stations being selected for inclusion via voluntary participation. The final analytic sample consisted of 400 valid responses which was adequate for stable bootstrapped estimation with respect to indirect effects in PLS-SEM.

### 3.3 Respondent profile and sample characteristics

Table 1 shows the respondents’ demographics and work characteristics in a short form. The sample is predominantly male and Saudi, with most participants aged between 25 and 34 or 35 and 44. There are ambulance drivers, EMTs/paramedics, supervisors/managers, and other clinical and support staff. EMTs/paramedics make up the largest group. Most of the people who answered have a bachelor’s degree and work for the government. The sample also shows that there are differences in EMS and ambulance-driving experience, with a lot of people from the mid- and long-tenure groups. Furthermore, respondents are allocated across various workload conditions regarding weekly working hours, night-shift frequency, and missions per shift, thereby reflecting the diversity in operational exposure and work demands within the Saudi EMS context.

**Table 1:** Demographic profile of respondents (*n* = 400).

Main categories	Sub-categories	Frequencies	Percentages %
Gender	Male	320	80.00
	Female	80	20.00
Age	<25 years	35	8.75
	25–34 years	170	42.50
	35–44 years	117	29.25
	45–54 years	63	15.75
	55+ years	15	3.75
Nationality	Saudi	348	87.00
	Non-Saudi	52	13.00
Education	Diploma	90	22.50
	Bachelor’s	229	57.25
	Master’s	58	14.50
	Doctorate/MD	10	2.50
	Other	13	3.25
Job role (driving-eligible groups)	Ambulance driver	70	17.50
	EMT/Paramedic	187	46.75
	Supervisor/Manager	63	15.75
	Other clinical/support	80	20.00
Employment sector	Government	284	71.00
	Private	77	19.25
	Military	31	7.75
	Other	8	2.00
EMS experience	<1 year	26	6.50
	1–3 years	83	20.75
	4–6 years	91	22.75
	7–10 years	109	27.25
	>10 years	91	22.75
Ambulance driving experience	<1 year	38	9.50
	1–3 years	85	21.25
	4–6 years	114	28.50
	7–10 years	95	23.75
	>10 years	68	17.00
Weekly working hours	<40 h/week	33	8.25
	40–48 h/week	199	49.75
	49–60 h/week	132	33.00
	>60 h/week	36	9.00
Night shifts (per month)	0	2	0.50
	1–4	82	20.50
	5–8	220	55.00
	9–12	86	21.50
	≥13	10	2.50
Missions per shift	1–2	26	6.50
	3–4	126	31.50
	5–6	148	37.00
	7–8	79	19.75
	≥9	21	5.25

## 4 Results

### 4.1 Evaluating the reflective measurement model

The first step for the reflective measurement model assessment is to test the internal consistency reliability and the convergent validity. Cronbach’s alpha has been typically used as a measure of the internal consistency reliability, with values higher than 0.70 generally seen as acceptable. As Cronbach’s alpha has some limitations, composite reliability (CR) is typically preferred, while CR values that lie between 0.70 and 0.90 indicate acceptable internal consistency reliability (47). Convergent validity was evaluated utilizing outer loadings and the average variance extracted (AVE). Outer Loadings should be greater than 0.708 and if the loadings between 0.40 and 0.70 must be retained when their removal does not improve CR (Composite Reliability) or AVE (Average Variance Extracted) values (48, 57). Thus, Table 2 shows indicator reliability, internal consistency reliability and convergent validity for the study constructs and their dimensionalities.

**Table 2:** Reliability and Convergent Validity of First-Order and Second-Order Composites.

Measures	*λ*	VIF	M	SD	SK	KU
Perceived stress (PS) (*α* = 0.926, CR = 0.937, AVE = 0.600)						
PS1	0.807	2.371	2.922	1.424	0.111	−1.309
PS2	0.774	2.050	2.960	1.435	0.060	−1.324
PS3	0.784	2.108	2.945	1.431	0.102	−1.324
PS4_R	0.773	2.056	3.035	1.452	−0.051	−1.352
PS5_R	0.812	2.363	3.072	1.441	−0.052	−1.335
PS6	0.779	2.145	2.980	1.412	−0.023	−1.319
PS7_R	0.770	2.132	3.000	1.393	0.017	−1.298
PS8_R	0.757	2.014	3.053	1.418	−0.019	−1.320
PS9	0.728	1.800	2.945	1.413	0.039	−1.307
PS10	0.759	2.019	2.962	1.400	0.040	−1.282
Cognitive failures (CF) (*α* = 0.766, CR = 0.865, AVE = 0.680)						
Cognitive failures—Memory (Me) (*α* = 0.896, CR = 0.920, AVE = 0.659)						
CF1	0.797	2.062	2.970	1.400	0.026	−1.295
CF2	0.807	2.056	2.913	1.439	0.063	−1.362
CF3	0.847	2.354	3.075	1.437	−0.101	−1.328
CF4	0.793	1.957	2.980	1.421	0.025	−1.329
CF5	0.835	2.307	3.002	1.390	−0.044	−1.270
CF6	0.788	1.920	3.005	1.380	0.008	−1.244
Memory	0.806	1.544	2.991	1.145	−0.099	−1.072
Cognitive failures—Attention (At) (*α* = 0.889, CR = 0.923, AVE = 0.751)						
CF7	0.851	2.198	3.035	1.423	−0.041	−1.305
CF8	0.865	2.300	3.035	1.426	−0.020	−1.336
CF9	0.879	2.471	2.998	1.408	−0.023	−1.283
CF10	0.870	2.464	3.065	1.441	−0.054	−1.310
Attention	0.832	1.611	3.033	1.234	−0.078	−1.185
Cognitive failures—Action (Ac) (*α* = 0.902, CR = 0.927, AVE = 0.719)						
CF11	0.849	2.404	3.083	1.413	−0.115	−1.302
CF12	0.835	2.324	3.050	1.419	−0.046	−1.290
CF13	0.839	2.229	3.035	1.412	−0.068	−1.312
CF14	0.849	2.335	3.118	1.405	−0.128	−1.281
CF15	0.867	2.498	3.027	1.406	−0.044	−1.289
Action	0.836	1.525	3.063	1.196	−0.168	−1.142
Emotional exhaustion (EX) (*α* = 0.930, CR = 0.943, AVE = 0.704)						
EE1	0.855	2.806	2.933	1.390	0.054	−1.249
EE2	0.836	2.555	3.018	1.399	0.024	−1.286
EE3	0.840	2.519	3.055	1.415	−0.029	−1.285
EE4_R	0.845	2.581	2.960	1.394	0.049	−1.278
EE5	0.820	2.327	3.013	1.406	0.005	−1.307
EE6	0.841	2.569	2.938	1.417	0.069	−1.312
EE7	0.836	2.556	3.002	1.426	0.011	−1.311
Risky driving (RD) (*α* = 0.815, CR = 0.878, AVE = 0.643)						
Speeding (SP) (*α* = 0.889, CR = 0.923, AVE = 0.750)						
SP1	0.890	2.593	3.000	1.437	−0.010	−1.350
SP2	0.856	2.116	3.020	1.439	−0.076	−1.310
SP3	0.873	2.501	3.002	1.385	−0.022	−1.263
SP4	0.845	2.301	3.115	1.446	−0.122	−1.341
Speeding	0.765	1.593	3.034	1.236	−0.045	−1.173
Rule violations (RV) (*α* = 0.876, CR = 0.915, AVE = 0.729)						
RV1	0.874	2.484	2.913	1.425	0.087	−1.307
RV2	0.837	2.094	2.935	1.416	0.068	−1.316
RV3	0.866	2.222	3.015	1.423	0.010	−1.316
RV4_R	0.837	1.988	3.042	1.470	−0.074	−1.379
Rule Violations	0.830	1.858	2.976	1.224	0.020	−1.170
Inattention/distraction (IN) (*α* = 0.890, CR = 0.923, AVE = 0.751)						
IN1	0.849	2.266	2.930	1.441	0.052	−1.352
IN2	0.879	2.390	2.970	1.412	−0.011	−1.296
IN3	0.876	2.457	2.897	1.417	0.060	−1.285
IN4	0.862	2.319	2.958	1.453	0.025	−1.386
Inattention/Distraction	0.813	1.808	2.939	1.240	0.008	−1.236
Driving while fatigued (DF) (*α* = 0.887, CR = 0.930, AVE = 0.815)						
DF1	0.895	2.492	2.953	1.421	0.032	−1.303
DF2	0.908	2.717	2.913	1.419	0.066	−1.290
DF3	0.905	2.463	2.880	1.432	0.171	−1.282
Driving While fatigued	0.797	1.576	2.915	1.286	0.089	−1.186

*λ* = outer loading; VIF = variance inflation factor; M = mean; SD = standard deviation; SK = skewness; KU = kurtosis. PS = Perceived Stress; CF = Cognitive Failures (second-order); Me = CF–Memory; At = CF–Attention; Ac = CF–Action; EX = Emotional Exhaustion; RD = Risky Driving (second-order); SP = Speeding; RV = Rule Violations; IN = Inattention/Distraction; DF = Driving While Fatigued. Items ending “_R” are reverse-coded.

The results presented in Table 2 provide strong evidence for the reliability and convergent validity of the reflective measurement model. All the retained indicators loaded satisfactorily on their respective constructs with outer loadings of above 0.728 and therefore, more than the recommended criteria (47). Furthermore, all the study constructs and dimensions exceeded the satisfactory internal consistency reliability (47), with Cronbach’s alpha values ranging from 0.766 to 0.930, and composite reliability values from 0.865 to 0.943, respectively. The AVE values fell between 0.600 and 0.815, and all exceeded the minimum threshold of 0.50, indicating that convergent validity was supported (48). Similarly, the VIF values were below critical limits, suggesting that multicollinearity was not of concern. These results overall confirm that the reflective measurement model shows sufficient reliability as well as convergent validity, and it is appropriate for further investigating discriminant validity and relationships in the structural model.

### 4.2 Measurement model evaluation

The global model fit assessment provided additional validation for the proposed model’s adequacy. Table 3 shows that the saturated model had an SRMR value of 0.047 and the estimated model had an SRMR value of 0.057. Both of these values were below the recommended threshold of 0.08, which means that the model fit was acceptable. Likewise, the NFI values for the saturated and estimated models were 0.912 and 0.913, respectively, which is higher than the usual cutoff of 0.80. We also looked at the discrepancy measures d_ULS and d_G to get more evidence about how well the model fits overall. These findings indicate that the model demonstrates adequate fit and is suitable for further structural model assessment (49, 50).

**Table 3:** Model fit indices for the saturated and estimated models.

Model Fit	Saturated model	Estimated model
SRMR	0.047	0.057
d_ULS	0.660	0.965
d_G	0.211	0.213
Chi-square	492.913	487.991
NFI	0.912	0.913

SRMR = standardized root mean square residual; d_ULS = squared Euclidean distance; d_G = geodesic distance; NFI = Normed Fit Index. Acceptable model fit is indicated by SRMR < 0.08 and NFI > 0.80 (49).

According to Table 4, the reflective first-order measurement model showed good discriminant validity. First, the Fornell–Larcker criterion states that the square root of the average variance extracted (AVE) for each construct was greater than its correlations with the other constructs. This means that each construct shared more variance with its own indicators than with other latent variables (51). The diagonal values were between 0.775 and 0.903, but the inter-construct correlations were always lower. Second, the HTMT ratios were all below the recommended threshold, which was between 0.188 and 0.657. This is more proof that the constructs are different from each other in real life (48, 49). The descriptive statistics also showed that the mean scores ranged from 2.915 to 3.062 and the standard deviations ranged from 1.103 to 1.288. This means that there was a moderate amount of variation between the answers. All of these results together show that the first-order reflective constructs are valid and give a good reason to move on to the evaluation of the second-order measurement model.

**Table 4:** Reflective measurement first-order: discriminant validity (Fornell–Larcker and HTMT) and descriptive statistics.

Construct	M	SD	PS	Me	At	Ac	EX	SP	RV	IN	DF
PS	2.987	1.103	**0.775**	0.438	0.487	0.433	0.530	0.188	0.264	0.250	0.318
Me	2.991	1.147	0.402	**0.812**	0.601	0.554	0.406	0.242	0.353	0.277	0.325
At	3.033	1.236	0.444	0.538	**0.866**	0.591	0.310	0.250	0.379	0.232	0.372
Ac	3.062	1.198	0.399	0.499	0.530	**0.848**	0.411	0.404	0.388	0.378	0.514
EX	2.988	1.182	0.493	0.371	0.284	0.378	**0.839**	0.385	0.387	0.404	0.430
SP	3.034	1.238	0.171	0.218	0.224	0.367	0.356	**0.866**	0.629	0.581	0.483
RV	2.976	1.225	0.238	0.314	0.336	0.348	0.351	0.556	**0.854**	0.657	0.593
IN	2.939	1.242	0.229	0.253	0.208	0.343	0.370	0.518	0.581	**0.867**	0.608
DF	2.915	1.288	0.290	0.293	0.331	0.461	0.394	0.430	0.524	0.540	**0.903**

Diagonal = √AVE; below diagonal = correlations; above diagonal = HTMT; M = mean; SD = standard deviation. PS = perceived stress; Me = CF–memory; At = CF–attention; Ac = CF–action; EX = emotional exhaustion; SP = speeding; RV = rule violations; IN = inattention/distraction; DF = driving while fatigued. The bold values represent the diagonal √AVE values (Fornell–Larcker criterion). Values below the diagonal are correlations, and values above the diagonal are HTMT ratios.

Then, it was necessary to assess the second-order measurement model. After adequate discriminant validity had been established, the second-order model was evaluated on a higher-order-construct basis after ensuring that the higher-order construct had indeed been properly indexed (48).

Table 5 shows that the second-order reflective measurement model had good discriminant validity. The Fornell–Larcker criterion states that the square root of the AVE for each construct was higher than its correlations with the other constructs. This shows that each construct was different from the others (51). Moreover, all HTMT values were beneath the suggested threshold, varying from 0.332 to 0.593, thereby reinforcing discriminant validity (48, 49). The descriptive statistics indicated that the mean scores varied from 2.966 to 3.029, with standard deviations between 0.985 and 1.182. These results show that the second-order measurement model is good enough and give us a good reason to move on to the structural model assessment and test the proposed hypotheses.

**Table 5:** Reflective measurement second-order: discriminant validity (Fornell–Larcker and HTMT) and descriptive statistics.

Construct	M	SD	PS	CF	EX	DF
Perceived stress (PS)	2.988	1.103	**0.775**	0.593	0.530	0.332
Cognitive failures (CF)	3.029	0.985	0.500	**0.825**	0.492	0.584
Emotional exhaustion (EX)	2.988	1.182	0.495	0.416	**0.839**	0.522
Driving while fatigued (DF)	2.966	1.000	0.294	0.471	0.457	**0.802**

Diagonal = √AVE (Fornell–Larcker); below diagonal = correlations; above diagonal = HTMT; M = mean; SD = standard deviation. The bold values represent the diagonal √AVE values (Fornell–Larcker criterion). Values below the diagonal are correlations, and values above the diagonal are HTMT ratios.

### 4.3 Hypotheses testing

The bootstrapping procedure in Smart-PLS was used to assess the structural model, to test our hypotheses. The purpose of this step is to test the significance, direction and magnitude of the direct and indirect associations between perceived stress, cognitive failures, emotional exhaustion, and risky driving controlling for the effects of control variables. Table 6 presents the path coefficients, *t*-values, *p*-values and decisions for all hypothesized relationships (48).

**Table 6:** Hypotheses testing results.

Hypothesis/control	Path	*β* (O)	*t*	*p*	Decision
Direct effect					
H1	PS → RD	−0.054	1.067	0.286	Not supported
H2	PS → CF	0.500	13.668	<0.001	Supported
H3	PS → EX	0.495	12.680	<0.001	Supported
H4	CF → RD	0.361	7.828	<0.001	Supported
H5	EX → RD	0.329	7.096	<0.001	Supported
Indirect effect					
H6a	PS → CF → RD	0.181	6.558	<0.001	Full mediation
H6b	PS → EX → RD	0.163	6.039	<0.001	Full mediation
Control effect					
C1	Age → RD	−0.040	0.863	0.388	Not significant
C2	Driving experience → RD	−0.023	0.532	0.594	Not significant
C3	Weekly working hours → RD	0.030	0.712	0.476	Not significant
C4	Night shifts/month → RD	−0.065	1.647	0.100	Not significant
C5	Night shifts/month → EX	−0.002	0.055	0.956	Not significant

*R*^2^ adjusted for the endogenous constructs was CF = 0.249, EX = 0.241, and RD = 0.301. *p*-values were obtained via bootstrapping in SmartPLS; values shown as 0.000 are reported as *p* < 0.001. Full mediation is inferred because both specific indirect effects are significant, whereas the direct effect (PS → RD) is non-significant.

The direct effect of perceived stress on risky driving was negative but not statistically significant (*β* = −0.054, *t* = 1.067, *p* = 0.286), indicating that H1 was not supported. Conversely, perceived stress exerted significant positive effects on cognitive failures (*β* = 0.500, *t* = 13.668, *p* < 0.001) and emotional exhaustion (*β* = 0.495, *t* = 12.680, *p* < 0.001), thereby corroborating H2 and H3. Cognitive failures significantly influenced risky driving (*β* = 0.361, *t* = 7.828, *p* < 0.001), while emotional exhaustion also exhibited a notable positive impact on risky driving (*β* = 0.329, *t* = 7.096, *p* < 0.001), thereby validating H4 and H5. The indirect relationships showed that perceived stress had a significant effect on risky driving through cognitive failures (*β* = 0.181, *t* = 6.558, *p* < 0.001) and through emotional exhaustion (*β* = 0.163, *t* = 6.039, *p* < 0.001). This supports H6a and H6b and shows full mediation. With regard to the control variables, age (*β* = −0.040, *p* = 0.388), driving experience (*β* = −0.023, *p* = 0.594), weekly working hours (*β* = 0.030, *p* = 0.476), night shifts per month on risky driving (*β* = −0.065, *p* = 0.100), and night shifts per month on emotional exhaustion (β = −0.002, *p* = 0.956) were all not significant.

**Figure 1:**
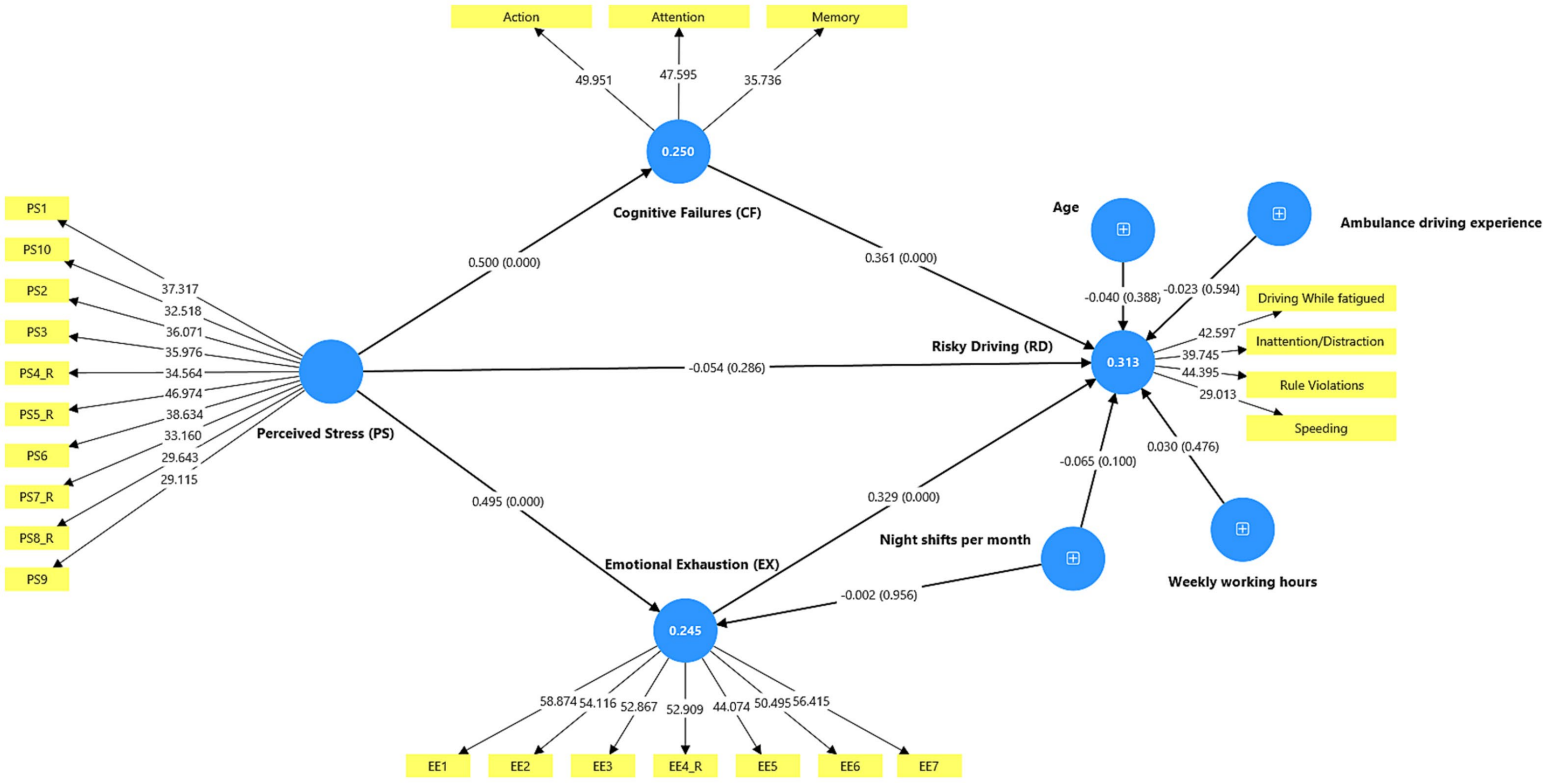
Structural model results.

Figure 1 shows the structural model and how well the endogenous constructs explain things. The adjusted *R*^2^ values show that the model accounted for 24.9% of the variance in cognitive failures, 24.1% of the variance in emotional exhaustion, and 30.1% of the variance in risky driving. These numbers show that the model was able to explain the main endogenous variables being studied at a level that was acceptable.

## 5 Discussion

In contrast to H1, perceived stress did not exhibit a significant direct impact on risky driving. A prior review of studies on this topic showed that switching from stressed to non-stressed conditions was associated with improved driving performance, which makes the current non-significant direct effect appear more nuanced rather than wholly contradictory (30, 31). However, other evidence has indicated that work-related stress is highly predictive of burnout and related depletion outcomes in emergency driving contexts (3, 5). Through the lens of transactional stress appraisal theory, this pattern hints at the notion that viewing demands as stressful does not always quickly become unsafe driving unless that appraisal somehow changes the psychological or emotional functioning of the driver. Similarly, from a conservation of resources perspective, stress might not lead to a behavioral effect unless it first depleted cognitive and affective resources that are necessary for safe driving performance. Likewise, from a conservation of resources perspective, stress may not exert a direct behavioral effect unless it first drains the cognitive and affective resources required for safe driving performance. A likely explanation is that professional training, emergency-driving protocols, and the strong sense of responsibility for patient safety in the Saudi EMS context may buffer the immediate stress–behavior link. In other words, EMS personnel may continue to regulate their driving effectively under perceived stress as long as key self-regulatory resources remain intact. In the current sample, therefore, perceived stress does not appear to directly influence risky driving but rather indirectly through more proximal cognitive and affective processes.

Supporting H2, the results demonstrated that perceived stress significantly and positively affected cognitive failures (*β* = 0.500, *p* < 0.001). This finding is broadly consistent with previous research. Previous studies have indicated that perceived stress is linked to higher cognitive failures, subjective cognitive complaints, and poorer cognitive performance across time (32, 33). This also corresponds to evidence suggesting that increased stress is associated with worse working memory performance and self-regulation under challenging work environments (35, 36). Additionally, studies in high-pressure care environments have found that heavy workload and stressful demands of work correlate with increased cognitive failures (34). This outcome aligns closely with transactional stress appraisal theory, which posits that when individuals evaluate situational demands as overwhelming their coping capacity, attentional control and cognitive monitoring are likely to decline. Finally, it is consistent with the conservation of resources theory since continuous stress denotes lack of mental resources necessary for attention, working memory and regulation of actions. However, this consensus is not universal: some evidence suggests that the detrimental effect of stress on cognition may depend upon contextual factors [in particular task demands, situational intensity and available coping resources (52)]. One possible explanation for this effect may be due to perceived stress in the EMS context draining attentional control, working memory, and executive regulation during time-urgent/multitasking/information overload scenarios in nature, thereby increasing the chance of cognitive failures occurring while on-duty would not contribute significantly at all.

In support of H3, perceived stress was also significantly and positively affected emotional exhaustion (*β* = 0.495, *p* < 0.001). This finding is in agreement with previous studies indicating that burnout and emotional exhaustion are increased among health professionals who report a higher level of perceived stress (38, 39). This conclusion is also consistent with ambulance-related evidence that has found stress to be a relevant psychological burden in emergency driving work, negatively relating to burnout-related outcomes (3, 5). Theoretical reasoning suggests that it is a relatively strong fit with conservation of resources theory, which suggests the mechanism through which continued exposure to challenging environments lowers levels of valued emotional resources such as sympathy and empathy resulting in their eventual depletion leading to negative work outcomes including emotional exhaustion. This is also consistent with transactional stress appraisal theory, as repeated appraisals that demands of duty exceed available coping resources will be met with prolonged emotional strain, and not solely momentary pressure. In the EMS context, repeated emergencies, trauma exposure, shift work, and sustained operational pressure may intensify the emotional costs of stress. Accordingly, the present result supports the view that perceived stress functions as an upstream affective burden that gradually erodes emotional energy in safety-critical work settings.

Supporting H4, cognitive failures significantly and positively affected risky driving (*β* = 0.361, *p* < 0.001). This finding is broadly in line with evidence linking cognitive failures to distracted driving, aggressive driving, and driving incidents (12, 13, 53). It is also consistent with earlier work associating cognitive failures with driving errors, lapses, and violations, especially through deficits in attention regulation and concentration (10, 11). Similar evidence from occupational driving settings shows that cognitive failure predicts slips, mistakes, and deliberate or unintended violations (42). This result, at least from a theoretical standpoint, serves to clarify the behavioral significance of stress-related cognitive depletion. Drivers who perceive themselves to be overloaded, feel safe when they are able to appraise and inhibit in time, but these individuals exhibit impaired information processing (how we process information), which transactional stress appraisal theory explains. Reduced cognitive resources makes less maintenance of safe behavioral regulation possible according to conservation of resources theory. The present result thus aligns with previous work in suggesting that cognitive failures are not merely incidental mental lapses, but a proximal behavioral mechanism through which unsafe driving emerges. A plausible reason for this consistency is that, in EMS work, driving occurs under time pressure, urgency, and high cognitive load, making attentional lapses and monitoring failures more consequential for driving safety (18). Thus, cognitive failures appear to operate as an important proximal determinant of risky driving during duty.

In line with H5, emotional exhaustion also significantly and positively affected risky driving (*β* = 0.329, *p* < 0.001). This finding is broadly in line with evidence indicating that emotional exhaustion diminishes the self-regulatory resources essential for vigilance, judgment, and safe driving behavior. There is direct evidence that emotional exhaustion is linked to driving anger, which is linked to violations, aggression, and near misses (14). Studies on professional drivers indicate that burnout leads to driving errors and fatigue-induced unsafe driving (15–17). This new finding reinforces the conservation of resources explanation, as emotionally depleted personnel may have fewer emotional and self-regulatory reserves at their disposal to sustain vigilant attention and drive with discipline. It also adheres to transactional stress appraisal theory by demonstrating that once stressful demands are appraised as overwhelming repeatedly, their clout may be behaviorally manifested in the form of emotional depletion. In EMS work, repeated emotional and operational strain may weaken alertness, emotional control, and behavioral restraint, thereby contributing to hazardous driving. Thus, emotional exhaustion appears to be not only a distress outcome but also a mechanism through which stress-related resource loss is translated into unsafe duty-driving behavior.

Confirming H6a and H6b, perceived stress significantly and positively affected risky driving via both cognitive failures (*β* = 0.181, *p* < 0.001) and emotional exhaustion (*β* = 0.163, *p* < 0.001), and the two indirect paths showed full mediation, respectively. This pattern is broadly in line with prior research suggesting that job stressors often influence unsafe driving through intermediate depletion mechanisms rather than through a direct behavioral path alone. Significantly, this is where the data best supports the two tentpole theories. Transactional stress appraisal theory helps us understand why stress becomes consequential when EMS personnel soon interpret operational demands as surpassing available coping resources, resulting in disruption to cognitive clarity and emotional regulation. This explanation is further supported by the conservation of resources theory, which posits that cumulative exposure to such demand has an accumulative draining-effect on cognitive and affective resources leading directly to risky driving behavior. The significant path from PS → CF → RD indicates that, due to its disruptive effect on attentional control, working memory, and action regulation, stress promotes risky driving because it increases cognitive failures (12, 13, 32, 33, 53). Simultaneously, the pathway PS → EX → RD highlights that stress also promotes risky driving behavior by depleting affective energy and self-regulation capacity, resulting in emotional exhaustion (14, 16, 17, 38, 44). The full-mediation finding is especially critical from a theoretical standpoint because it indicates that perceived stress alone is not the ultimate determinant of unsafe behavior. Instead, stress operates on the cognitive and emotional pathways that both theories suggest. A possible explanation for this pattern is that in EMS work, stress might not always be manifested through unsafe driving as long as the personnel possess cognitive control and emotional regulation; but when these proximal resources become impaired due to stress, risky driving behavior becomes more pronounced. The full-mediation pattern thus provides an explanation of why perceived stress influenced risky driving indirectly as opposed to directly. Hence, in Saudi EMS personnel, perceived stress appears to affect risky driving not directly but rather through two separate but parallel cognitive and affective paths.

Finally, the control variables failed to be significant, suggesting that age, ambulance driving experience, weekly working hours and night-shift frequency did not substantially change the main structural relationships. As such, this pattern further supports the theoretical prominence of proximal psychological mechanisms over background demographic features in accounting for risky driving within the current model. In overall, the findings suggest that cognitive failures and emotional exhaustion are the main mechanisms through which perceived stress leads to dangerous driving on duty.

## 6 Theoretical and practical implications

The study makes multiple theoretical contributions. First, it contributes to the occupational health and driving-safety literature by demonstrating that perceived stress was not associated with risky driving directly but through two parallel significant pathways: cognitive failures and emotional exhaustion. This adds nuance to the stress–safety relationship by suggesting that stress only becomes behaviorally relevant when it gets translated into proximal cognitive failures and emotional exhaustion. Second, the findings bolster transactional stress appraisal theory and conservation of resources theory relevance to safety-critical EMS settings. Following along these lines, stress seems to matter not simply as a generalized strain experience but as the way in which obligation demands drain cognitive control and emotional resources and increases risky driving behavior. Third, the study combines cognitive and affective pathways within one model that is more comprehensive than models based on direct effects of stress alone to explain increased risky driving specifically among EMS personnel. Fourth, the study broadens to context-specific paradigms with evidence from Saudi Arabia, a setting that continues to be under-represented in the EMS safety and occupational health literature, while demonstrating that primary explanatory pathways were more pronounced than those of demographic and work-schedule ascertainment included.

Practically, the findings indicate that EMS organizations should not view risky driving simply as an issue of traffic compliance or technical driving skill. Instead, risky driving should again be regarded as an occupational health and psychosocial safety issue. Since the relationship between perceived stress and awareness was non-significant and only indirect effects through cognitive failures and emotional exhaustion were significant, effectiveness of interventions may be increased when it comes to attentional functioning and emotional depletion rather than stress alone. This suggests that routine monitoring for cognitive lapses, exhaustion and symptoms of stress should be implemented in all EMS agencies; fatigue-risk-management, recovery opportunities and workload organization should be strengthened; supervision with psychological support should be put in place; brief interventions on attentional control, stress management to enhance emotional recovery could potentially provide benefit. In high-pressure ambulance operations, pre-shift or during-shift fit-to-drive checks may also be beneficial. In general, promoting EMS driving safety might need to safeguard more than just driving behavior; it may also need to protect the cognitive and emotional functioning that interacts with safe governing duty driving.

## 7 Limitations and future research directions

This study, similar to other survey-based research, possesses numerous limitations. The data were obtained through a cross-sectional self-report design, which constrains causal inference and may be influenced by common method bias or social desirability. Second, while the sample was sufficient, it was exclusively composed of EMS personnel from Saudi Arabia, potentially constraining the generalizability of the findings to other emergency service systems or cultural contexts. Third, the study concentrated on two mediating mechanisms—cognitive failures and emotional exhaustion—while neglecting other pertinent organizational behavior variables, including safety climate, perceived organizational support, leadership style, job demands, job resources, and coping strategies. Fourth, self-reported behavior was used to measure risky driving instead of objective driving records or observational indicators. Subsequent research could employ longitudinal or time-lagged methodologies, incorporate multi-source or objective driving data, and examine additional organizational, cognitive, and affective mechanisms within more extensive EMS and cross-national samples to enhance the robustness and generalizability of the results.

## 8 Conclusion

This study contributes to the growing literature on risky driving among EMS personnel by demonstrating, first, that perceived stress does not directly predict risky driving but rather acts via two important parallel mediators: cognitive failures and emotional exhaustion. These findings, in the context of Saudi EMS, suggest that stress becomes consequential for behavior when it erodes attentional control and working memory and self-regulatory capacity on which we have relied through effects such as affective depletion under sustained duty pressures. So risky driving during ambulance duty seems to be influenced less by stress in and of itself, and much more by the cognitive and emotional impairment that it brings. By incorporating cognitive and affective pathways into a single model, the study identifies cognitive failures and emotional exhaustion as the primary mechanisms through which perceived stress manifests in unsafe driving. These findings indicate that EMS driving safety may potentially benefit from not only focusing on traffic law compliance and technical driving skills, but also enhancing organizational efforts to minimize cognitive lapses related to stress and emotional exhaustion while on duty.

## Data Availability

The original contributions presented in the study are included in the article/supplementary material, further inquiries can be directed to the corresponding author.
